# Pathways to Modern Family Planning: A Longitudinal Study on Social Influence among Men and Women in Benin

**DOI:** 10.1111/sifp.12145

**Published:** 2021-02-08

**Authors:** Susan Igras, Sarah Burgess, Heather Chantelois‐Kashal, Mariam Diakité, Monica Giuffrida, Rebecka Lundgren

**Affiliations:** ^1^ Susan Igras, Mariam Diakité, Institute for Reproductive Health, Center for Child and Human Development Georgetown University Washington DC USA; ^2^ Sarah Burgess, Camber Collective Seattle WA USA; ^3^ Heather Chantelois‐Kashal, Independent Consultant; ^4^ Monica Giuffrida, Bill & Melinda Gates Institute for Population and Reproductive Health Johns Hopkins Bloomberg School of Public Health Baltimore MD USA; ^5^ Rebecka Lundgren, Division of Infectious Disease and Global Public Health, Department of Medicine, Center on Gender Equity and Health University of California San Diego CA USA

## Abstract

Despite improvements in family planning (FP) knowledge and services in West Africa, unmet need for FP continues to grow. Many programs apply a demographically and biologically driven definition of unmet need, overlooking the complex social environment in which fertility and FP decisions are made. This longitudinal, qualitative cohort study captures the changing nature of FP need, attitudes and behaviors, taking into account life context to inform understanding of the complex behavior change process. Purposively sampled, 25 women and 25 men participated in three rounds of in‐depth interviews over 18 months. Analyses used a social network influence lens. Findings suggest alignment of six foundational building blocks operating at individual, couple, services, and social levels is essential to meet FP need. If one block is weak, a person may not achieve met need. Women and men commonly follow five pathways as they seek to fulfill their FP need. Some pathways achieve met need (determined users, quick converters), some do not (side effect avoiders), and some do not lead to consistent FP outcomes (male‐priority decision makers, gender–egalitarian decision makers). Findings clarify the role of social determinants of FP and offer insight into program approaches informed by user typologies and return on program investments.

## BACKGROUND

Survey data from 52 low‐ and middle‐income countries indicate that women's preferred family size is often smaller than their actual family size (Sedgh et al. 2016), suggesting that women face barriers to achieving their reproductive intentions (Sedgh et al., 2016, 9). Moreover, literature indicates that unmet need for family planning (FP) remains high and continues to grow (Sedgh et al., 2016; Cleland et al., 2015; Casterline and El‐Zeini 2014; Machiyama and Cleland, 2015; Rossier et al., 2014). Nearly half of currently married women of reproductive age report not wanting to have a child soon, yet fewer than one in 10 use modern contraception (Sedgh et al., 2016). Benin is no exception. DHS studies indicate that unmet need has increased from 21 percent of women aged 15–49 in union in 1996 to 32 percent in 2017/2018 (INSAE, 2019). Modern contraceptive use over the same time has grown from 3 percent to 12 percent (INSAE, 2019). One‐third of women of reproductive age report discontinuing a method in the 12 months before the most recent DHS survey; while almost none report changing to a different method in the same period (INSAE, 2019). Understanding socionormative factors at play in Benin may shed light on why unmet need for FP is rising in Benin.

The obstacles women face managing their fertility are complex and multifaceted. In a recent review, the most common factors influencing the use of contraceptives in sub‐Saharan Africa included fear of side effects, male partner disapproval, sociocultural and normative beliefs around fertility, women's education and employment, and partner communication (Blackstone et al., 2017). Studies have also indicated that it is not lack of access to modern contraception but rather infrequent sex and concerns regarding side effects or other health risks that drive contraceptive nonuse (Moreira, Ewerling, Barros, & Silveira, 2019; Sedgh & Hussain, 2014). Gender role expectations and other social factors related to FP communication and decision‐making play essential roles in method adoption and continuation (Belizzi et al. 2015). These social and behavioral factors, in turn, interact with individual pregnancy motivations and risk‐benefit calculations that change over time and with life situations (Speizer & Lance, 2015). Increasingly, researchers recognize that the categorization of pregnancies as planned or unplanned does not do justice to the complex, dynamic nature of fertility experiences (Arteaga, Caton, & Gomez, 2019). Nevertheless, current program paradigms rarely focus on the dynamic social sphere in which FP decisions are made.

Findings from the baseline survey of the Tékponon Jikuagou intervention project shed some light on how these issues play out specifically in Benin (https://www.thecompassforsbc.org/filteredsearch/tekponen%2520jikuagou). Data indicated that 36 percent of women reported that it was not acceptable to talk about FP in public; 11 percent of women reported discussing FP with their husbands in the last year; and egalitarian couple decision‐making was uncommon (IRH and CRAD, 2014). As efforts to achieve the Sustainable Development Goals gain momentum, better understanding of underlying social and individual factors that influence unmet need could improve social and behavior change efforts. To address this knowledge gap, we explored the following research questions to analyze social and other influences on women and men's decisions and behaviors vis‐à‐vis FP use, using a social influence lens and social network theory (Igras et al., 2016) to attend to the context of changing households and community dynamics:

1. What assets and barriers influence people to act (or not) on unmet need for FP?
2. Are there patterns or typical pathways that women and men take as they seek to meet their FP needs?
3. How might understanding of these pathways provide insight into FP policy and programming?

We use a longitudinal, cohort approach to investigate the processes involved in FP behavior change among women and men over 18 months, leading to a typology of FP users based on understanding the dynamic nature of FP need, attitudes and behaviors, life context, and the role of enabling factors and barriers supporting a change process. We conclude with a discussion on policy and program design implications.

## METHODS AND MATERIALS

### Sampling and Data Collection

We conducted three rounds of semistructured, in‐depth interviews in January 2013, October 2013, and September 2014. Interviews took place with 25 women of childbearing age (18–49 years, married/in union) and 25 men married to women of childbearing age in 12 villages in the Couffo Department in southwest Benin. Retention rates were high, 94 percent of Round 1 participants interviewed at the third and final round.

Participants were purposively selected by project extension workers to yield a study population representing individuals with differing social network influence and FP need status. Cohort participants were not couples, but all were married/in union. Extension workers chose three villages (18 in total) in the Health Zones under their supervision and identified four to five participants per village. Participants were identified without consideration of whether they were engaged in Tékponon Jikuagou. Twelve of the 18 possible villages were selected in two Health Zones. Extension workers then visited potential participants, completed a brief interview that defined their baseline social network and unmet need statuses, and enrolled 50 participants.

Interview guides were informed by formative research and a literature review and designed to generate discussion of themes relevant to FP use. As the interview rounds progressed, we modified the interview guides to probe deeper into emerging themes. Professional interviewers conducted interviews, visiting the same respondents over three rounds. Participants and interviewers were the same gender to facilitate dialogue. The transcripts suggest that some interviewers and respondents developed trusting relationships over time that may have led to franker discussion.

Ethical clearance was obtained from the Georgetown University Institutional Review Board and the Comité d'Éthique de la Recherche at the Institut des Sciences Biomédicales Appliquées in Benin. Interviewers obtained signed consent before the first interview and oral consent before subsequent interviews. Interviews took place in Adja or Fon and were transcribed into French by interviewers.

### Analysis

Our analysis consisted of three phases. In the first phase, we used coding (Corbin and Strauss, 1990) to identify essential factors related to unmet need that changed over the course of the study. We organized transcripts according to gender, site, and need status and coded with pre‐established and emergent codes using AtlasTi 7. Code themes included social networks, FP need, FP knowledge, couple relationships, sources of information and support, individual and reference group attitudes about gender, FP, and child spacing, experience with health services, and engagement with FP interventions. Our interest focused on understanding the role of social influence in FP, including social networks (Sinai et al. 2017, Igras et al., 2016). Accordingly, we analyzed spousal influence, service factors, and social influence from family, peers, and leaders on FP use, as well as how participants, in turn, influence others in their communities. We also focused on understanding conditions under which need statuses changed over time. Thus, analysis of need status included not only those with a biological risk of pregnancy but also those whose *beliefs* about risk put them at risk of pregnancy, to reflect the range of motivations to achieve met need. This broadened definition allowed us to consider, for example, people who believed they had met their FP need and were protected from pregnancy, but who in reality were still at risk of pregnancy (having perceived no need, below). The shaded rows in Table 1 represent people with met need or no need, that is, with no risk of pregnancy according to the standard definition. Unshaded boxes show those who fit a broadened definition, those at risk of pregnancy while believing they are protected.

**TABLE 1:** Broadened definition of unmet need incorporating actual and perceived statuses

Actual no need	Realizes correctly that pregnancy is not possible, and uses no method, for example, in menopause, had a hysterectomy, pregnant, or not having sexual relations
Actual met need	Realizes pregnancy is possible and uses a modern method
Perceived met need	Realizes pregnancy is possible and uses a traditional method in the belief of being protected
Perceived no need	Believes pregnancy is not possible, even though at biological risk, for example, breastfeeding, postpartum, infrequent sex
Perceived unmet need	Realizes that pregnancy is possible, but does not use a method for various reasons, for example, partner disapproval, sterility, or poor health

SOURCE: Sinai et al. (2017).

In the second phase, we employed matrix analysis (Corbin and Strauss, 1990) to examine the dynamics of unmet need with essential factors identified in Round 1. Matrices categorized participants’ experiences and change processes in each relevant theme over interview rounds, allowing identification of factors most likely to drive change. Couple dynamics, for instance, emerged as a dynamic factor. In contrast, the concept of child‐spacing changed little across the interview rounds. Factors that showed little variation were deemed less critical for further analysis and modeling. Six crucial factors—the *Building Blocks to FP Met Need*—emerged that form a person's foundation or asset base to achieve met need. We then used matrices to examine participant experiences across the six building blocks related to their FP need and use status. Matrices had different focuses, including the influence of participants’ social networks and the processes of receiving and sharing (diffusing) new ideas, attitudes, and behaviors. Subquestions guiding analysis within each building block were organized by participants’ current situation and family context.

In the third phase, we used grounded theory and content analysis (Strauss and Corbin, 1994) to organize respondents into pathways leading to met/unmet need and to understand commonalities within identified pathways. We categorized each participant according to need status at each interview round, analyzing groups of participants with similar trajectories (e.g., men who began the study with unperceived unmet need and ended with met need using a modern FP method). Seven participants (three women and four men) had no need for FP or followed distinct, outlier pathways and were excluded. Eight participants (two women and six men) had two pathways and are included twice in the FP Pathways analysis (51 cases). After establishing trajectory groups, their shared characteristics and important factors, we compared groups that began with the same need status but diverged over the study period (e.g., comparing the group of men who end with met need to men who begin and end with unperceived unmet need). Next, the team used code reports and matrices to analyze each identified factor to understand the subdynamics that either enable, create barriers, or appear to have no significant influence on FP use, disaggregating findings by gender.

It became apparent that shared factors affect people's ability to meet their FP needs and that individuals follow common pathways to navigate these factors, resulting in met or unmet FP need. To understand these pathways, we used grounded theory and content analysis to analyze participants’ experience of the building blocks of FP met need, identifying the most influential factors on FP trajectories and placing participants into groups based on shared key themes in their FP journeys. The analysis and comparison of these pathways form the “FP Pathways” section of the article.

A nine‐member analysis team processed high volumes of data over several years, strengthening the analysis through different perspectives of US‐ and Benin‐based staff. Routine team check‐ins alongside efforts to check and improve inter‐rater reliability were employed in all data analysis rounds.

The study described in this article was a complement to implementation research on a social network intervention, Tékponon Jikuagou, to reduce unmet need for FP. Although the cohort study was exploratory and not intended to evaluate the intervention, it did occur in intervention villages where the project increased the exposure of many participants to FP information. Thus, while this study considers the influence of Tékponon Jikuagou (and other FP interventions) on cohort participants as they define and seek to meet their FP needs, it does not assess the effect of the intervention on FP outcomes.

## RESULTS

### Characteristics of Study Participants

Table 2 summarizes participant characteristics. Divided equally by sex, participants were of Fon and Adja ethnicities; all married/in union, began the study with varying FP need statuses; ranged in age from their twenties to forties, and had various levels of engagement with FP interventions.

**TABLE 2:** Characteristics of cohort study participants

	Men		Women	
	(n = 25)		(n = 25)	
Age (years)				
20–29	7		7	
30–39	11		16	
40+	7		2	
Ethnicity				
Adja	18		19	
Fon	7		6	
Engagement in Tékponon Jikuagou				
Yes	21		20	
No	4		5	
FP need status	Round 1	Round 3	Round 1	Round 3
Actual need				
Met need	6	12	7	13
No need	6	6	7	10
Unmet need	3	1	5	1
Perceived need				
Met need	6	3	8	5
No need	3	0	1	0
Unmet need	9	5	9	5

### Building Blocks of FP Met Need

**FIGURE 1:**
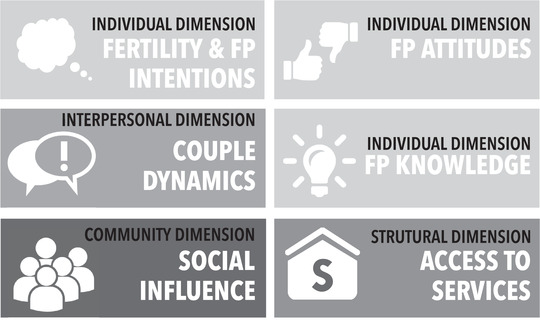
Building blocks of family planning met need

The initial round of analysis indicated that study participants required a strong foundation to obtain FP information and services and use a method correctly over time. Six *building blocks of FP met need* emerged. These blocks, which manifest as enabling factors or barriers, reflect the complex personal and collective factors in study communities in southwest Benin. Building blocks of FP met need encompass individual, interpersonal, structural, and community dimensions that influence knowledge, attitudes, normative expectations, and behavior within and across social levels (Figure 1). The six blocks are mutually reinforcing with alignment usually required for a person to meet their FP need. That is, it took only one absent or weak block to derail a person's efforts to achieve their FP intention. The blocks often changed direction (enabling / barrier) over the 18 months of the study, influencing consistent and correct use of contraception over time. Blocks are described below.

*Fertility and FP Intentions*. Nearly all participants indicated they wanted to avoid pregnancy during one or more of the three interview rounds; roughly one‐third of participants desired a pregnancy at some point during the study. Some had strong intentions to avoid pregnancy over 18 months; many other participants had uncertain or changing fertility intentions.

*Family Planning Attitudes* were defined as (1) the extent to which FP is considered beneficial for family life; (2) the perceived merits of birth spacing; (3) the perceived qualities of men and women who use FP; and (4) the perceptions of side effects, based on reality or myth.

*Family Planning Knowledge* refers to *awareness* of (1) a range of FP methods, (2) where to acquire services and information, (3) how to use different methods, and (4) understanding of their potential side effects.

*Couple Dynamics* encompass (1) a participant's ability to discuss and make decisions related to FP with her/his partner, (2) partner concordance or discordance with FP decisions, and (3) the degree of shared or individualized responsibility for seeking and using a method.

*Access to Services* was defined multidimensionally as (1) the ability to visit a health center to obtain FP, (2) the availability and affordability of FP products, (3) quality of services, and (4) the perception of whether a woman needs her husband's consent to obtain FP.

*Social Influence* refers to the web of social relationships that surround individuals (social networks) that influence behavior. Social influence offers negative or positive reinforcement for individual behaviors and attitudes and can fortify or change existing dynamics and assets when cognitive, emotional, practical and material support is provided.

These six blocks emerged as critical and distinct factors that directly influenced FP use. Contextual factors—such as gender or socioeconomic status—were also of vital importance to participants' identity and experiences. Yet, gender and socioeconomic status did not emerge as discrete factors influencing the evolution of FP use in this particular cohort. Instead, they were woven throughout the six building blocks.

### FP Pathways

Across the cohort, over 18 months, we identified patterns in the relationships among the six building blocks and demographic factors, such as age, gender, and household composition. We refer to these patterns as *FP pathways*. Each pathway is temporal and relational and may be viewed as a cluster of factors, unified around a common theme, which creates the context that individuals navigate as they define and seek to meet their FP needs. Profiles for each pathway are described below, named to indicate the defining characteristic of people in each pathway. Table 3 shares a snapshot of an illustrative respondent for each pathway.

**TABLE 3:** Snapshots of illustrative respondents for different pathways

Ending in met need	Determined Users (40+ years, 10 children, Adja, traditional religion, agriculturalist, secondary education) In the past, Daniel and his two wives hesitated to use FP due to concerns about side effects and gender dynamics. By the time the study had begun, however, all three had overcome these fears and were longtime users of modern contraception. Daniel's social network—especially his in‐laws—was encouraging of FP use, and participation in Tékponon Jikuagou further strengthened his FP knowledge and capacity. During the second round of interviews, Daniel described this evolution toward more positive FP attitudes and the reasons for his FP use: “*Even before [Tékponon Jikuagou] came, my first wife had gone for FP. I did not like it at all, and we argued about it. But now, I myself have understood and [FP] allows us to avoid repeated illness of the children, and we save a lot of money*.” (R2) By the end of the study, both wives were continuing to use modern contraception.
	Quick Converters (30–39 years, fewer than six children, Adja, Christian, agriculturalist, primary education) Sabine had a six‐month‐old and used the rhythm method. Though she was very interested in using a modern method, distance and limited FP options at her local health center were barriers to use. She became a Tékponon Jikuagou catalyzer (group discussion leader), which equipped her with basic knowledge of the FP options available at her health center. After becoming involved, she spoke to her husband, and they happily began using an implant: “*My husband and I regularly talk about the children we want and how to space those births. Often during [conversation], we say that 6 children are enough for me, the wife, and that we should think about their future… I really appreciated the proposal of my husband, and some days later I went to use the implant*.” (R2)
Not achieving met need	Side Effect Avoiders (30–39 years, four children, Fon, Christian, agriculturalist, no education) Lisette and her husband decided that they only wanted four children. However, they had only boys and wanted a girl, so she gave birth to their fifth child, a boy, just before her last interview. In the past, they had used withdrawal to space their pregnancies. They now searched for a different method to use long‐term. Despite some exposure to the intervention through a community group, Lisette lacked precise information on modern methods. At the same time, she witnessed close friends suffer method side effects. Consequently, she consistently expressed significant fear of side effects, and at the end of the study, Lisette and her husband only considered using a traditional method: “*I like the implant, but as people say that it has side effects and that [it] can also get lost in the body, that discourages me. [My husband] asked me if he was only going to continue with withdrawal.”* (R3)
No consistent FP end point	Male‐Priority Decision Makers (25–29 years, three children, Fon, Christian, agriculturalist, no education) Chantal and her husband began discussing FP more openly once she began participating in cohort interviews. Still, she believed that he must take the lead, and she could never use a FP method in secret. Chantal started the study with three children and wanted only four. Her husband initially agreed because he could marry another wife and have children with her, but he then changed his mind and said that she could use FP only after she had six children. Although they agreed to use a modern FP method, he chose to continue using withdrawal, and she had an unintended pregnancy. At the end of the study, she was still waiting for him to select and use a modern method: “*There is no change [in my FP use] because my husband has not made up his mind since you arrived to go for FP. Without his input, I can't do it*.” (R3)
	Gender–Egalitarian Decision Makers (30–39 years, 10 children, Adja, traditional religion, agriculturalist, secondary education) Although Marcel's history of FP use is unclear, his collaboration with his wife on FP issues and birth spacing is apparent from the beginning of the study. He and his wife learned about FP together, sharing information and perspectives. His involvement as a Tékponon Jikuagou catalyzer helped the couple to consider a range of methods: “*Today, this discussion between my wife and I [on FP] is more frequent because of the new knowledge we have of these methods thanks to the arrival of Tékponon Jikuagou and also because of the cost and difficulties of the current life*.” (R2) Despite negative community attitudes toward FP and FP users, they made the decision together to use an implant and were very satisfied with it.

We next discuss the five main FP pathways that study participants followed during the 18‐month study. Two pathways, *Determined Users* and of *Quick Converters*, led to met need. One pathway, *Side Effect Avoiders*, led to unmet need. Two pathways, *Male‐Priority Decision Makers* and *Gender–Egalitarian Decision Makers*, did not lead to consistent FP outcomes.

Participants were categorized in the pathway that most strongly influenced their FP trajectory over the three interview rounds. However, FP need is complex, and multiple themes shaped some participants’ trajectories. Thus, eight participants (two women and six men) were categorized into two pathways, and both pathways are included in our discussion. Table 4 summarizes sociodemographic information of participants in each pathway. Note that each pathway contains relatively equal proportions of women and men, except for the two gender‐influenced pathways.

**TABLE 4:** Sociodemographic characteristics of pathway members (n = 51)

		Total	Sex		Age (in years) at beginning of study			Number of children at beginning of study^a^			
		In pathway	Women	Men	20–29	30–39	40+	0–4	5–6	7+	Unspecified
Ending in met need	Determined Users	15	8	7	5	7	3	7	5	3	0
	Quick Converters	7	3	4	2	5	0	2	2	2	1
Not achieving met need	Side Effect Avoiders	8	3	5	1	6	1	4	1	1	2
No consistent FP end point	Male‐Priority Decision Makers	11	7	4	4	3	4	6^a^	3^a^	0	2^a^
	Gender–Egalitarian Decision Makers	10	3	7	3	6	1	6	1	2	1

^a^ Not all participants clearly stated how many children they had, particularly for men in polygamous unions, who sometimes referred to their children with one wife and sometimes all children born of all of their wives. Our best guess based on transcripts is noted above.

Below, we discuss each of the five pathways, shedding light on (1) openness of a pathway's members to change in FP behavior; (2) critical barriers to and enablers to meeting FP need over time; (3) social network actors who might or did influence their FP attitudes and behaviors; and (4) FP use trajectory over the study period. Table 5 provides a summary of each pathway's enabling factors and barriers to achieving met need. Social network members influencing FP behaviors are also listed.

**TABLE 5:** Comparison over 18 months of building block influences on family planning pathways, by enabling factors and barriers (ranked in order of importance), and by people who influence pathway members (ranked in order of relative stated importance as FP‐influential within social networks)

			Beginning of study		End of study		
		Pathway	Enabling factors	Barriers	Enabling factors	Barriers	Key FP‐influencers
Pathways leading to	Met need	Determined Users	Fertility and FP intentions FP attitudes Social network (support)	Social network (FP knowledge and attitudes) FP knowledge Service access	Fertility and FP intentions FP attitudes Couple dynamics	None	Parents of both spouses (especially the husband) Husband's brother Friends
		
		Quick Converters	FP attitudes	FP knowledge Social network (FP attitudes and support)	Fertility and FP intentions FP attitudes FP knowledge Social network (support)	None	Health workers Village health team envoys Religious leaders Parents Siblings Friends of both spouses
		
	Unmet need	Side Effect Avoiders	Fertility and FP intentions	Social network (FP attitudes) FP attitudes (specific to side effects)	Fertility and FP intentions Social network (support)	FP knowledge Access to services	Husband's mother Wife's parents Husband's brother Wife's sister, aunt, or uncle Friends Health workers FP community groups
		
	No consistent end point	Male‐Priority Decision Makers	FP attitudes	FP knowledge Couple dynamics	FP attitudes FP knowledge Social network (support)	Couple dynamics FP knowledge	Husband's family Wife's friends or aunt Health workers Religious leaders
		
		Gender–Egalitarian Decision Makers	Fertility and FP intentions Couple dynamics	FP knowledge Social network (FP attitudes) Service access	FP attitudes Social network (support)	Social network (FP attitudes)	Parents and friends of both spouses Health workers Religious leaders

*Determined Users* exemplify a pathway of FP uptake or continued use characterized by positive FP attitudes and intentions, strong proactiveness, and an ability to address challenges, including, for four women, covert method use. For the most part, *Determined Users* were already convinced of the benefits of FP at the time of the first interview, and strong fertility and FP intentions and sense of self‐efficacy drove them to use a modern method of FP. This pathway is illustrated by Dora (all named participants in this article have been given pseudonyms). A long‐term user of pills, Dora, had positive FP attitudes and high self‐efficacy from Round 1. By the last interview, her husband had married another woman, and Dora aimed to change her FP method without his knowledge: “*I don't want my husband to know about it, I'm thinking about using the injectable… As he has become polygamous, he doesn't take care of me anymore. I'm the only one who will take care of myself and support the children*.” (R3)

At the beginning of the study, positive fertility and FP intentions, FP attitudes, and social network support provided an enabling foundation for FP use. However, determined users also noted weaknesses in social network knowledge and attitudes, FP knowledge, and service access. By the end of the study, participants had overcome those weaknesses and described a supportive or neutral FP foundation across building blocks.

One such participant was Goussou. Although Goussou and his wife used contraception throughout the study, he noticed by the third interview that community discussions on FP and its benefits seemed to be replacing those centered on the importance of having many children, which was a noted weakness in his foundation: “*In the last 12 months, there's not a place you go where you don't hear about FP, so FP is a good thing, it gives the woman more freedom*.” (R3)

Our analysis of social networks identifies health workers, parents of both spouses (but especially the husband), the husband's brother, and friends as promising/viable network actors to transmit messages and positively influence *Determined Users. These participants were consequently able to attain high rates of modern FP use across the study period. Over half were using a modern method of FP when the study began; all used a modern method at some point during the study; all who needed FP were using a modern method at the time of the last interview*.

*Quick Converters demonstrate rapid adoption of modern methods of FP once minor barriers are addressed*. These participants began with strong FP attitudes and, by the end of the study, had overcome small barriers in FP knowledge and social network support. Though they sometimes confronted FP stigma and myths/beliefs about methods, *Quick Converters* also developed active and often positive social network engagement over time that helped address concerns about FP. Despite community members discouraging her from using FP at Round 1, for example, Yvette, a young mother of 6 children, was able to overcome this largely unsupportive environment and get an implant due in part to the positive influences of her husband, co‐wives, and even her parents: “*[I don't want to get pregnant] because I only have a few‐days‐old baby. Plus, my parents are very worried about me, and they want me to stop; they don't want me to get pregnant again*.” (R1)

Furthermore, knowledge improvements enabled participants to transition from traditional to modern methods of pregnancy prevention. This is exemplified by Paul. By the third interview, Paul had gained knowledge on the different types of modern contraceptive methods and he and his wife chose to use an implant: “*The information and advice [Tékponon Jikaugou] gave on FP methods motivated me to bring my wife to the hospital for the implant. As an influential person, I advise people on FP methods by my example*.” (R3)

Of note, All *Quick Converters* played an active role in the Tékponon Jikaugou program, either as participants or group leaders, implying that program engagement played a crucial role in rapid conversion. Health workers and village extension agents, religious leaders, and parents, siblings, and friends of both spouses were identified as people with whom they spoke about FP or who influenced FP decisions and who could be mobilized as influencers in future programming. *All Quick Converters started the study with perceived or unperceived unmet need, mostly using withdrawal or a combination of withdrawal, condoms, and a rhythm method. By the time of the final interview, all used an implant, injections, or oral contraceptives*.

*Side Effect Avoiders* demonstrate a FP pathway dominated by difficult‐to‐address emotions and experiences around side effects. These participants began with strong fertility and FP intentions. Although FP attitudes improved for half of these participants over 18 months, challenges persisted in access to services, couple dynamics, FP knowledge, and social network attitudes, all serving as barriers to modern method use. Although most participants across pathways voiced *concern* about side effects of modern FP methods, *fear* that FP would cause serious health problems, particularly sterility, profoundly limited the acceptability of modern methods for this group, despite expressed commitment to limiting or spacing pregnancy.

Although he stated in the first interview that FP helps couples to space their children, for example, Aristide, a polygamous man with four wives, declared that he and his wives fear FP use as they believed it would cause infertility: “*We often hear people say that women no longer menstruate after using FP methods and that they no longer do so after childbirth. All of these things worry us*.” (R1)

Additionally, four of the spouses who had significant influence over FP decisions resisted FP use due to their fears. One of these was Mathieu. Despite wanting to use a modern method of contraception and space his children, Mathieu had unmet need for FP at the beginning of the study as his wife feared that using it would lead to her having future fertility problems: “*I don't use FP methods because my wife has difficulties getting pregnant… Even if I propose to use FP, she is afraid that it will cause her problems*.” (R1)

Thus, participants are included in this trajectory when their own or their spouse's intense fear of side effects prevented them from meeting their FP needs. Participants on this pathway were likely to say that they had not discussed FP with any community leader or health professional. Most said that they had discussed such issues with no one. (A few did say that health workers, religious leaders, or community group leaders had influenced their FP opinions.) *At the end of the study period, most participants in this pathway were still concerned about side effects and chose methods such as withdrawal, rhythm, abstinence, or condoms that they felt confident would not affect their health or fertility*.

*Male‐Priority Decision Makers* experienced a FP pathway most profoundly influenced by the husband's power over fertility and FP decision‐making. Four participants who followed this pathway were men who claimed the right to make final FP decisions, and six were women who either willingly granted their husbands this right or who felt compelled to do so to respect traditional gender norms and mitigate power dynamics. *Male‐Priority Decision Makers* began the study with ambiguous fertility intentions and strong FP attitudes. Over the study period, their social network support improved, while FP knowledge, couple dynamics, and for some service access, were persistent challenges. The extent of the power imbalance between partners varied, as did the extent of women's resistance to their partner's domination of FP decision‐making.

One exemplar of the male‐priority decision makers group was Marie Rose, a married woman who had recently given birth when she entered the study largely supported the decision‐making power of husbands over wives on matters related to fertility as this upheld a wife's traditional subservient role to her husband: “*No woman can use a FP method without her husband's consent… There is no point in being a wife if you and your husband don't understand each other; you can't get up and ask for advice behind his back*.” (R1)

Men in this group all described making decisions without seriously considering their wives’ perspectives. Despite describing how he and his four wives discuss the spacing of their children and their FP use, for example, Affisou also invoked traditional gender norms, where women without strong, masculine male protectors are susceptible to infidelity or engaging in sex work. When describing why men have the final say in fertility and FP decisions: “*The wife must always submit to her husband. Otherwise, she is said to look elsewhere or to prostitute herself*.” (R2)

Another man in the study, Francois, described intentionally impregnating his wife without her consent, again implying that fertility is an expression of masculinity for this group. Although restricted agency in FP decision‐making is a significant barrier to meeting FP needs, participants on this pathway had diverse FP trajectories. The husband's family, the wife's friends or aunt, health workers, or religious leaders were listed as people who did or might influence these participants’ FP decisions. *At the beginning of the study, roughly half began with unmet need (perceived or unperceived), and only one began with met need. By the end of the study, over half had achieved met need, while three still had unmet needs*.

*Gender–Egalitarian Decision Makers* demonstrate an FP pathway characterized by couple collaboration and mutual respect in FP decision‐making. As with *Male‐Priority Decision Makers*, the degree of gender equality decision‐making varied. However, men in this pathway described their wives as able to influence their opinions, engage in dialogue, and withhold agreement until both were satisfied with the final decision. Gerard, for example, expressed from the first interview that both members of a couple need to be implicated in FP discussions and decisions: “*The husband and wife must first agree on FP before they both go to a health worker*.” (R1)

Furthermore, both men and women stated that they were able to initiate discussion of FP and child spacing with their partners and conveyed satisfaction with the level of openness, frequency of discussion, and their ability to be heard. Efomo, a mother of three who wanted to wait before becoming pregnant with her last child, described initiating a discussion on FP use with her husband and together they agreed to use condoms: “*The day [that my husband] came home, the time was not favorable [for pregnancy], and I was thinking about how to tell him that the time was not favorable. When he came back to the room, and we finished eating, he makes gestures, and I know he is asking me for the bed tonight. When I saw those gestures, I called him and told him that I implored him to use the condom, that the time is not favorable [for pregnancy] and he accepted easily*.” (R3)

These participants began the study with strong fertility and FP intentions and positive couple dynamics. Their FP attitudes, knowledge, and social network support improved over the study period, while service access and social network attitudes were persistent barriers and they rarely discussed FP with others. *Participants on this pathway had diverse FP trajectories. Five began with unperceived unmet need and three with met need, and approximately equal numbers ended the study with unperceived unmet need, met need, and no need. Despite having significantly more collaborative partner relationships and stronger fertility intentions than participants in the Male‐Priority Decision Maker pathway, these participants made slightly less progress in meeting their FP needs*.

Looking across pathways, by Round 3 those whose pathways led to met need—*Determined Users* and *Quick Converters*—faced no significant barriers and enjoyed numerous enabling factors (Table 5). The block alignment and direction are less consistent with pathways that did not lead to met need or were inconsistent as trajectories, affirming the foundational nature of the Building Blocks construct.

## DISCUSSION

Six foundational elements—building blocks for FP met need—were defined through inductive analysis of transcripts of 50 cohort individuals, creating a framework of enabling factors and barriers that led to the development of the FP pathways discussed in this article. The alignment of these building blocks, which operated at individual, couple, community and structural levels, and reflected social network influences, helped individuals to achieve their met need.

Several factors emerged that contributed to the complexity of meeting FP need: Fears about side effects, for example, were central in *all* six blocks. Couple relations—particularly the desire to create peaceful homes—helped shape four blocks, including fertility intentions, FP attitudes, access to services, and social influence. Finally, stigma played a notable role in couple dynamics and social network influence.

Participants began the study with varied FP foundations (building block configurations) and life‐course situations, and followed five diverse pathways, whether to met or unmet need. Building blocks on normative and attitudinal aspects of FP were the most resistant to change, and barriers in those areas shaped pathways leading to unmet need. Contrarily, barriers related to support from crucial social network members, FP knowledge, and access to services were easier to overcome and featured in pathways to met need. As participants’ contexts and life‐course dynamics shifted over 18 months, so did the character of their FP pathways.

Each pathway offers different program opportunities and challenges. With an eye toward potential return on investment in FP programming, we asked the following: What was each pathway's openness to uptake of modern contraception? Who influenced FP decisions? What aspects of services created barriers to those intending to seek services?

*Determined Users* tend already to be modern method users. As their FP needs may shift over time, programs might focus on clarifying method side effects and options for switching methods, to maintain the program investment.

*Quick Converters* are highly open to change. If engaged with FP programs, method use will likely result (the “low hanging fruit” of potential users), and thus offer a quick return on investment. At the beginning of the study, they already had well‐articulated FP and fertility intentions, indicated readiness to change, were receptive to external influences such as health agents, and could almost immediately benefit from external program support to transition to modern method use. Program efforts to motivate *Quick Converters* to use modern methods would focus on building knowledge and self‐efficacy, facilitating an exploration of social and health consequences of modern FP use, challenging anti‐FP attitudes and beliefs within social networks.

*Gender–Egalitarian Decision Makers* could also yield high returns on program investment due to their positive FP attitudes and intention to avoid pregnancy and relatively homogenous barriers to FP uptake, such as consistent negative attitudes toward FP within their social networks. Programs might strengthen participants' self‐efficacy by offering models of satisfied FP users and improve the ease and quality of service access, including the availability of a wide range of FP method options. Interventions could create social network support by strengthening FP‐related interactions with parents and friends of both spouses, health workers, and religious leaders.

*Male‐Priority Decision Makers* might be a challenge to engage, with less security of return on investment given the comparatively high degree of group heterogeneity and the range of barriers to modern method uptake represented in this pathway. Given the breadth of weaknesses in their FP foundations, programs targeting *Male‐Priority Decision Makers* would need to work across multiple blocks. They might help to strengthen fertility and FP intentions and enhance couple communication, highlight the benefits of FP for the couple and the family, emphasize men's positive roles and rewards in using FP, and increase and clarify the range of available FP methods and service access options. Programs might also need to challenge harmful models of masculinity which position control over fertility as important for social status and standing.

*Side Effect Avoiders* represent perhaps the most challenging pathway to address, as it is more complicated than others. At the same time, programs focused on reaching *Side Effect Avoiders* would likely have spillovers to other pathways, due to the persistence of side effect fears as a barrier to met need (Sedgh et al. 2016; Cleland et al., 2015) and the vital role of social network validation of positive FP experiences in encouraging modern method uptake (Mumah et al., 2019; Igras et al., 2016; Gayan et al., 2007). Programs targeting *Side Effect Avoiders* would prioritize individual and social network barriers relating to fear of health and social consequences that were expressed to varying degrees in *all* six building blocks to met need. They might focus on strengthening self‐efficacy—particularly management of side effects—and improving access to and knowledge of the range of methods and means of changing methods if concerns arise. Countering myths about FP would be essential, especially around method use and infertility. This group defined cost barriers broadly. Unlike *Quick Converters* focused on short‐term costs of method use, *Side Effect Avoiders* hesitated to pay for methods whose ensuing health complications might result in further medical expenses over the years and lost labor due to poor health. Programs might opt to sequence influencers, for example, beginning with focused health worker interaction before focusing on influential people within the social networks of *Side Effect Avoiders*.

In summary, findings suggest that social and behavior change programs would benefit from tailoring strategies and materials to support the needs of each group. Prioritizing *Quick Converters* and *Gender–Egalitarian Decision Makers* might provide more rapid returns on investment than other pathways. *Determined Users* need support when they wish to switch methods but otherwise are self‐sufficient in meeting their FP need. A different type of program effort is likely necessary to gain returns with *Side Effect Avoiders* and *Male‐Priority Decision Makers*. *Side Effect Avoiders* represent the most challenging and possibly complex pathway to address programmatically. No easy return on investment is evident, and new program approaches are needed, beyond assuming that improving knowledge alone addresses deeply rooted fears. Tackling this pathway requires nuanced understanding of side effects concerns.

Interestingly, the gendered pathways were the most divergent and the least predictable in achieving FP need. Men and women in the *Gender–Egalitarian Decision Maker* pathway had lower rates of met need than those in the *Male‐Priority Decision Maker* pathway, seemingly because both members of egalitarian couples expressed great fear of gendered social stigmatization of FP users. In contrast, other considerations were the primary barriers for members of nongender–egalitarian couples. Thus, both men and women who engaged in gender–egalitarian relationships (likely deviating from the broader social norm) experienced greater empowerment within the couple dyad but greater disempowerment in the broader community, adversely affecting their FP outcomes. Greater nuance in our understanding of the relationship between gender norms and unmet need is required. These results indicate that on its own, positive couple communication is not enough to create an enabling FP foundation, and other factors such as discussions with peers and family are needed to help even collaborative couples to meet their FP needs. Future studies on unmet need may, therefore, expand program paradigms, as has been done by the International Center for Research on Women' informative exposition of barriers to women's reproductive control (McLeary‐Sills et al., 2012). Further work to explore male engagement paradigms is needed, using comprehensive and gender‐synthesized analyses of gender systems, and employing a less monolithic view of men and masculinities (Hook et al., 2018).

Finally, other factors not included in the pathway analysis reported here also shaped some (nine of 50) participants' FP intentions and use. For instance, two participants did not need FP due to strong pronatalist values reinforced by their social networks. Additionally, four women are excluded from the analysis because the structure of their household—living apart from their husbands due to labor migration—shaped their FP use. They consequently had low perceived and actual risk of pregnancy. These women fluctuated between no need and met need throughout the study and had less FP knowledge and social network engagement than most other participants. Such women (and their partners) highlight how FP dynamics are linked with macro‐economic trends and are an important group to consider in West Africa and other areas with high labor migration.

We note several study limitations. Although systematic efforts to ensure reliability occurred in all analytic phases, we recognize that the consistency of interpretation from multiple people working on matrices and other analyses could be an issue. Most participants’ drop out occurred in the third round, meaning some trajectories that could have informed pathways were not included. Finally, the cohort data are not couple data; there might have been different trajectories if couples’ data were used in the study. Similarly, the trajectories seen for people in union in this study might differ for young people and those who are not married/in union.

## CONCLUSION

This qualitative longitudinal research approach allowed a deep, nuanced understanding of the complexity of gender norms and the social contexts in which FP decisions take place, documenting changing need and the dynamic nature of individuals' FP use over time in ways that cross‐sectional, quantitative results cannot. These findings offer useful insights for FP programs seeking to address unmet need, including the importance of outreach strategies to help align the inter‐related building blocks that influence met need for FP. Understanding pathway typologies can inform the allocation of program resources.

There is a need to better contextualize gender analyses and male engagement in FP. Many FP studies and programs explicitly or implicitly frame FP as a women's issue, positing that women are the ones who bear the most physical, social, and economic consequences of an unplanned pregnancy. Such approaches may unintentionally marginalize men's experiences, roles, and considerations (Cleland et al., 2015). These results reveal the depth of men's experiences, roles, and concerns, and bring nuance to male engagement realities and possibilities. These epistemological and methodological biases also mask the complex interdependency between partners and how couples as a unit and as individuals may be socially and structurally disempowered from meeting their FP needs.

If we are to reach the Sustainable Development Goal of universal access to reproductive health by 2030, we must do a better job of taking into account the social environment in which fertility decisions are embedded and better address the relationality of women and men to achieve met need and sustained use of FP. Recognizing the dynamic, contextualized nature of unmet need can help programs address unmet need, gender, and other sociocultural considerations, while also providing accessible, high‐quality FP information and services.
